# *EjODO1*, a MYB Transcription Factor, Regulating Lignin Biosynthesis in Developing Loquat (*Eriobotrya japonica*) Fruit

**DOI:** 10.3389/fpls.2016.01360

**Published:** 2016-09-16

**Authors:** Jing Zhang, Hang Ge, Chen Zang, Xian Li, Donald Grierson, Kun-song Chen, Xue-ren Yin

**Affiliations:** ^1^College of Agriculture and Biotechnology, Zhejiang UniversityHangzhou, China; ^2^Zhejiang Provincial Key Laboratory of Horticultural Plant Integrative Biology, Zhejiang UniversityHangzhou, China; ^3^The State Agriculture Ministry Laboratory of Horticultural Plant Growth, Development and Quality Improvement, Zhejiang UniversityHangzhou, China; ^4^Plant and Crop Sciences Division, School of Biosciences, University of NottinghamLoughborough, UK

**Keywords:** *EjODO1*, lignification, loquat, MYB, transcriptional regulation

## Abstract

Lignin is important for plant secondary cell wall formation and participates in resistance to various biotic and abiotic stresses. Loquat undergoes lignification not only in vegetative tissues but also in flesh of postharvest fruit, which adversely affects consumer acceptance. Thus, researches on lignin biosynthesis and regulation are important to understand loquat fruit lignification. In loquat, a gene encoding an enzyme in the lignin biosynthesis pathway, *Ej4CL1*, was reported to be regulated by transcription factors, including *EjMYB1, EjMYB2, EjMYB8*, and *EjAP2-1*, knowledge of this process is still limited. With the aim of identifying novel transcriptional factors controlling lignin biosynthesis in loquat, the promoter of *Ej4CL1* was utilized to screen a cDNA library by yeast one hybrid assay. A novel R2R3 MYB, named *EjODO1*, was identified. Real-time PCR analyses indicated that *EjODO1* is highly expressed in lignified stems and roots. During fruit development, expression of *EjODO1* decreased along with the reduction of lignin content and became undetectable in mature ripe fruit. Thus, *EjODO1* is likely to be involved in lignification of vegetative organs and early fruit development but not in mature fruit or postharvest lignification. Dual-luciferase assay indicated that EjODO1 could trans-activate promoters of lignin biosynthesis genes, such as *EjPAL1, Ej4CL1*, and *Ej4CL5* and transient overexpression of *EjODO1* triggered lignin biosynthesis. These results indicate a role for *EjODO1* in regulating lignin biosynthesis in loquat which is different from the previously characterized transcription factors.

## Introduction

Lignin is a complex phenylpropanoid polymer that constitutes a vital component of plant secondary cell walls, and imparts ‘waterproofing’ capacity as well as mechanical strength, rigidity, and environmental protection. The classical building blocks of lignin polymer comprise *p*-hydroxyphenyl (H), guaiacyl (G), and syringyl (S) (Boerjan et al., 2003; Rogers and Campbell, 2004; Vanholme et al., 2010). Various enzymes are involved in the lignin biosynthesis pathway, and most of them have been targeted for down-regulation, generating plants with reduced lignin levels or altered ratio of lignin monomers (Nicholas and Clint, 2010; Vanholme et al., 2013).

In addition to the biosynthetic pathway, a complex network of transcription factors has been reported regulating lignin biosynthesis. MYB transcription factors have been widely investigated and *AtMYB58, AtMYB63*, and *AtMYB85* specifically and directly activated the genes encoding the monolignol biosynthetic enzymes by binding to a conserved AC element in their promoters, but not genes for the cellulose and hemicellulose biosynthetic pathways (Zhong et al., 2008; Zhou et al., 2009). Similar MYB transcription factors have also been reported in other plants, such as *PtMYB1* and *PtMYB4* in *Pinus taeda* (Patzlaff et al., 2003a,b), *PtoMYB216* in *Populus spp*. (Tian et al., 2013), and *ZmMYB31*in *Zea mays* (Fornalé et al., 2010). On contrast, *AtMYB4*, an active repressor, negatively regulated the expression *AtC4H* (Jin et al., 2000). *AtMYB7* and *AtMYB32*, homologs of *AtMYB4*, also repressed lignin biosynthesis genes, such as *AtPAL, AtC4H, At4CL1*, and *AtCOMT* (Jin et al., 2000; Preston et al., 2004; Fornalé et al., 2014).

Lignin accumulation in perennial woody trees but rarely occurs in flesh fruit and has mainly been reported in pear (stone cells, Lu et al., 2015; Yang et al., 2015), *mangosteen* (pericarp, Ketsa and Atantee, 1998; Kamdee et al., 2014), and loquat (Cai et al., 2006c). In loquat fruit, lignin content increasing during postharvest storage in flesh (edible layer), which was associated with an increase in firmness and reduction of juice yield (Cai et al., 2006b), subsequently adversely affecting fruit edible quality. More worse, loquat is chilling sensitive fruit, as flesh lignification of loquat fruit can also be accelerated by low temperature (0°C, Cai et al., 2006d). Chill injury induced lignification can be alleviated by low temperature conditioning (LTC), acetylsalicylic acid (ASA), 1-methylcyclopropene (1-MCP), methyl jasmonate (MeJA), heat treatment (HT), etc (Cai et al., 2006a,b,d; Cao et al., 2008; Xu et al., 2014). Due to the significance of lignification and the existence of numerous effective treatments, loquat fruit was widely used as a model fruit to understand flesh lignification.

Investigations of the mechanisms underlying loquat lignification have been at both the biochemical- and molecular- level. Analysis of most of the biosynthetic enzymes, including phenylalanine ammonia lyase (PAL), 4-coumarate: coenzyme A ligase (4CL) and cinnamyl alcohol dehydrogenase (CAD), showed a correlation with fruit lignin content (Cai et al., 2006c; Shan et al., 2008) and their coding genes have also been analyzed. *EjCAD1* was first identified as a key candidate in regulated chilling injury-related lignification (Shan et al., 2008); the expression of other genes, such as *EjCcoAOMT*, also showed some positive correlation with lignin content in loquat fruit (Liu et al., 2015) and *Ej4CL1* was significantly inhibited by LTC and HT, which indicated *Ej4CL1* also involved in loquat fruit chilling injury induced lignification (Unpublished data). Recently two *EjMYB* genes, i.e., *EjMYB1* and *EjMYB2*, were characterized (Xu et al., 2014) and shown to be capable of binding to the promoters of lignin biosynthetic genes, but they manifested opposite roles in regulating lignification, with *EjMYB1* acting as an activator and *EjMYB2* as a repressor (Xu et al., 2014). Subsequently, an AP2/ERF gene, EjAP2-1, was discovered to be able to interact with EjMYB at the protein level (Zeng et al., 2015) and *EjAP2-1* transcriptionally suppressed the *Ej4CL1* promoter, involving the EAR motif in *EjAP2-1* acting via *EjMYB* (Zeng et al., 2015). Meanwhile, *EjNAC1*, homolog of *AtVND6* and *AtVND7*, was also shown to act as a positive upstream regulator of loquat fruit lignification and could trigger lignin accumulation in tobacco leaves (Xu et al., 2014). All these results indicated that fruit lignification can be regulated by a range of transcription factors (Xie et al., 2016).

Interestingly, among the promoters of structural genes in the lignin pathway that were tested, changes in *Ej4CL1* promoter activity showed the most significant responses to these transcription factors when tested, including three EjMYB (Xu et al., 2014; Wang et al., 2016), EjAP2-1 (Zeng et al., 2015), EjHSF3 (Zeng et al., 2016), and EjNAC1 (Xu et al., 2015). These findings led us to conduct the present research leading to the identification of, a R2R3 type MYB transcription factor, *EjODORANT1* (*EjODO1*), which is a homolog of petunia volatile related *PhODO1* (Spitzer-Rimon et al., 2012) and was obtained as the result of yeast one hybrid screening by using the *Ej4CL1* promoter as bait. The regulatory mechanisms of *EjODO1* was analyzed using real-time PCR, dual-luciferase, and transient over-expression systems.

## Materials and Methods

### Plant Materials

Red-flesh loquat ‘Luoyangqing’ (LYQ) fruits were collected from an orchard in Luqiao (Zhejiang province, China) at six different stages during fruit development: S1, fruitlet, 60 days after full bloom (DAFB); S2, immature green, 75 DAFB; S3, mature green 90 DAFB; S4, breaker, 100 DAFB; S5, half ripe 108 DAFB; S6, fully ripe 115 DAFB. Fruit harvested at 115 DAFB reached commercial maturity. Fruit without visible disease and mechanical wounding were selected for the study and three replicates were set for all sampling points. To test tissue specificity of the expression of *EjODO1*, vegetative tissues, including roots, stems and leaves, were harvested from germinated seedlings from seeds of loquat fruit, flowers were harvested at full bloom from orchard trees. Samples for all materials were frozen in liquid nitrogen and stored at -80°C.

### RNA Extraction and Real-Time Quantitative PCR

Total RNA and cDNA for different tissues and developmental stages of loquat were prepared according to the protocol described by Zeng et al. (2015). For Real-time PCR, gene specific primers were designed using Primer3 (vision 0.4.0)^1^. The specificity of these primers was determined by examining the melting curve and product resequencing. All reactions were normalized using the Ct value corresponding to the loquat actin gene *EjACT* (Fu et al., 2012). Primers for *EjODO1* were as follow: forward *5′*- ATTCCCCAAGCAATGAGTCTCAG-*3′*; reverse *5′*- TGCTAAGCTATTCTCCTCCGTTGG -*3′*. Real-time PCR analysis was performed with a LightCycler 1.5 instrument (Roche) using a mixture (10 μl total volume) comprising 2 μl of 5 × LightCycler FastStart DNA Master^PLUS^ SYBR Green I Master Mix (Roche), 0.5 μl of each primer (10 μM), 1 μl of diluted cDNA and 6 μl PCR-grade H_2_O. The PCR conditions included an initial denaturation for 5 min at 95°C, followed by 45 cycles of 95°C for 5 s, 60°C for 5 s, and 72°C for 10 s, and completed with a melting- curve analysis program. Three biological replicates were included for each sampling point or tissue.

### Yeast One Hybrid Library Screening and Confirmation

Yeast one hybrid library screening was conducted with the Matchmaker^TM^ Gold Yeast One-Hybrid Library Screening System (Clontech, Mountain View, CA, USA). A cDNA library was constructed with different loquat tissues (including root, stem, leaf, flower stalk, pericarp, and pulp) and used as prey and the promoter of *Ej4CL1* was used as bait for library screening. The construct of the *Ej4CL1* promoter was described in our previous report (Xu et al., 2014). All colonies were sequenced and blasted to GenBank for annotation. In order to verify the interaction between transcription factors and *Ej4CL1* promoter, 11 full-length specific transcription factors (excluding previously characterized *EjMYB1, EjMYB2*, Xu et al., 2014; *EjMYB8*, Wang et al., 2016) were sub-cloned into pGADT7 vector, the corresponding pGADT7 clone and pGADT7 empty vector was, respectively, transformed into Y1HGold[pEj4CL1/AbAi]. AD-p53 was transformed into Y1HGold[p53/AbAi]. Yeast one-hybrid assays were conducted on *SD* medium with aureobasidin A and without leucine (*SD*/Leu + AbA) at 30°C for 3–5 days to test the interaction.

Alignment of *EjODO1* with R2R3 EjMYB in both loquat and *Arabidopsis* was performed using the neighbour-joining (NJ) method in ClustalX (v.1.81), and a phylogenetic tree was constructed with FigTree (v.1.3.1).

### Dual-Luciferase Assay

Dual-luciferase assay was carried out to investigate the transactivation activity of transcription factors to target promoters. Full-length *EjODO1* was amplified and inserted into *Not*I and *Spe*I sites of pGreen II 0029 62-SK vector, while pGreen II 0800-LUC carrying promoters of lignin biosynthesis were constructed by Xu et al. (2014). All constructs were electroporated into *Agrobacterium tumefaciens* GV3101, using Gene PulserXcell^TM^ Electroporation Systems (Bio-Rad). Dual luciferase assays were performed according to our previous reports (Zeng et al., 2015). The empty vector mixed with the promoters was set as 1 and the analysis was carried out with at least three replicates.

### Transient Over-Expression Analysis of *EjODO1* in *Nicotiana tabacum* leaves

In order to verify the role of *EjODO1* in the regulation of lignin biosynthesis (based on present results) and fragrance biosynthesis (*ODO* genes were previously linked to volatiles, Verdonk et al., 2005), a transient expression transformation system was adopted in *N. tabacum* leaves.

*Agrobacterium* cultures with *EjODO1* or empty vector pGreen II 0029 62-SK (SK) were suspended in the same infiltration buffer used for dual luciferase assay and OD_600_ were adjusted to 0.75. Target gene (*EjODO1*) and negative control (SK) were infiltrated on two separate sides of the fifth true leave of *N. tabacum*. Five days after infiltration, tissue from each of the infiltrated leaves was taken for lignin and volatile compounds analysis.

Lignin content of tobacco leaves was measured according to the methods described by Xu et al. (2014). Data were expressed on a fresh weight basis, and all measurements were done in triplicate.

Volatiles were collected by placing 0.2 g frozen tissues powder into a 4 ml headspace vial containing 1 ml saturated sodium chloride solution and 50 μl 1-hexanol (0.1%, v/v) was added as an internal standard. The samples were incubated at 40°C for 30 min with continuous agitation (600 rpm) after fully vortexing for a few seconds. A SPME fiber coated with 50/30 μm Divinylbenzene/Carboxen/Polydimethylsiloxane (DVB/CAR/PDMS; Supelco Co., Bellefonte, PA, USA) was used for the extract of volatiles under the same conditions (40°C, 600 rpm). Volatile compounds were determined as described by Shen et al. (2016). The internal standards were used for compensating differences between samples, and the abundance of each volatile was calculated based on its peak area.

## Results

### Yeast One Hybrid Screening for Proteins Interacting with the Promoter of *Ej4Cl1*

**FIGURE 1:**
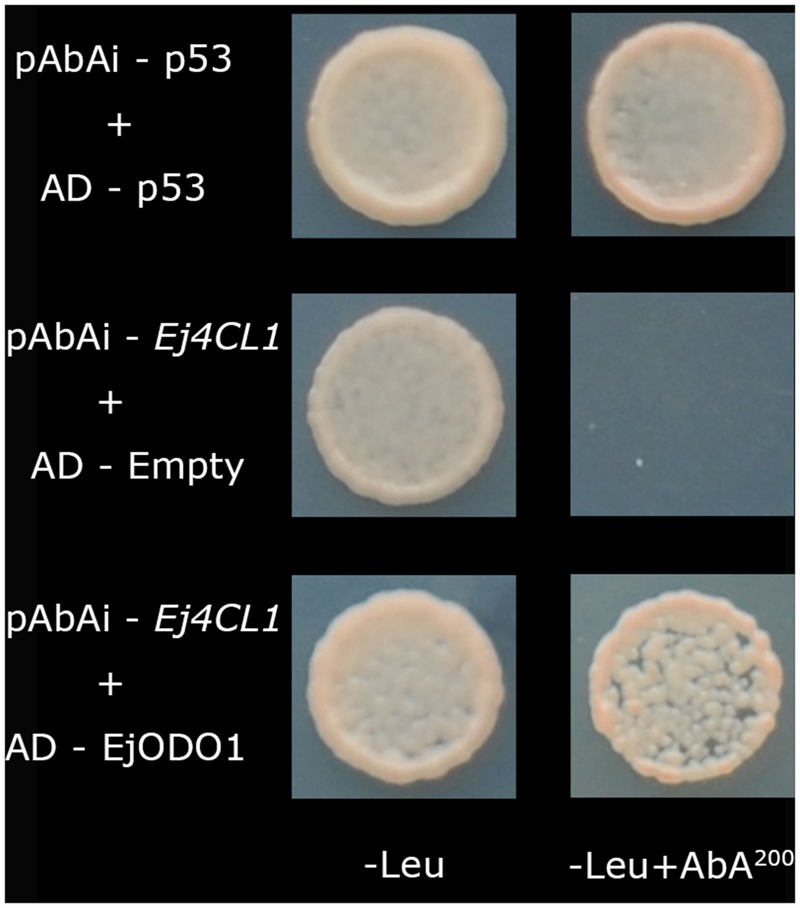
**Protein–DNA interaction between EjODO1 and the promoter of *Ej4CL1* using yeast one hybrid analysis.** Interaction was determined on SD medium lacking Leu in the presence of aureobasidin A (-Leu + AbA^200^). AD-p53 and pAbAi-p53 were used as a positive control; AD-empty and pAbAi-pEj4CL1 were used as a negative control.

In our previous studies, *Ej4CL1* was shown to be regulated by *EjMYB1, EjMYB2, EjMYB8, EjAP2-1*, and *EjHSF3* (Xu et al., 2014; Zeng et al., 2015, 2016). In order to obtain further information about the transcriptional regulatory mechanism controlling lignin biosynthesis in loquat, yeast one-hybrid screening was employed to look for novel transcription factors, using the *Ej4CL1* promoter as a bait. A total of 173 PCR products were obtained (data not shown), among which were 11 putative transcription factors including three *MYBs* (the previously characterized *EjMYB* genes were disregarded), three Zinc finger proteins, three *AP2/ERFs*, one *bHLH*, and one *NAC* (**Supplementary Figure S1**). However, only *EjODO1* (Genbank no. KX347550) showed high binding affinity for *Ej4CL1* promoter, unlike the other 10 putative transcription factors (**Figure 1**; **Supplementary Figure S1**).

### Phylogenetic Analysis of *EjODO1*

**FIGURE 2:**
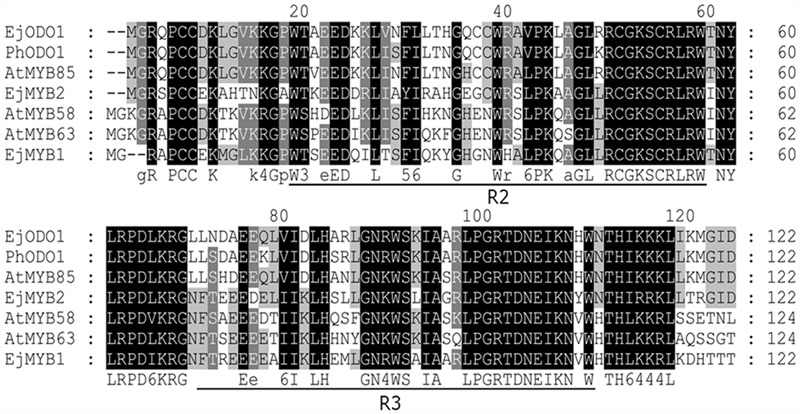
**Alignment of amino acid sequences of the R2R3 domains of *EjODO1, PhODO1*, and other MYBs.** Identical amino acid residues were shaded in black, and similar in gray. The R2 and R3 MYB conserved amino acid sequence among the different genes were underlined and refer to two repeats of the MYB DNA-binding domain.

**FIGURE 3:**
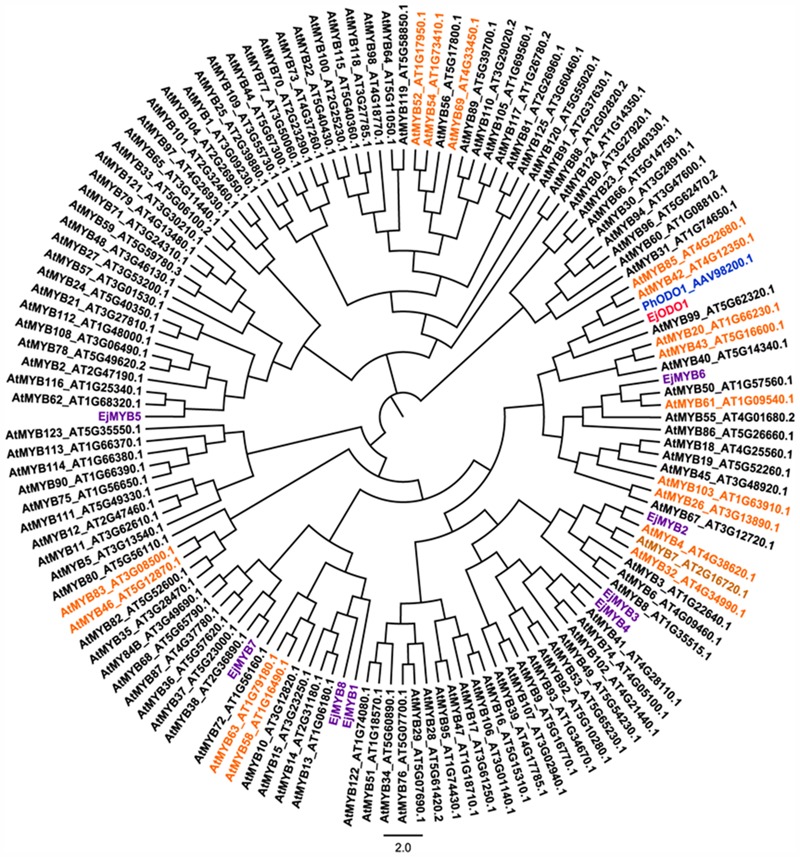
**Phylogenetic analysis of EjODO1, PhODO1, and R2R3 MYB members from loquat and *Arabidopsis*.** Fragrance biosynthesis related PhODO1 was highlighted in blue, the MYBs that involved in the regulation of lignin biosynthesis or secondary wall metabolism in *Arabidopsis* were indicated in orange and previously reported loquat EjMYB were highlighted in purple. Full length MYB protein sequences from *Arabidopsis* were downloaded from The *Arabidopsis* Information Resource (TAIR), and their accession number were embedded in the diagram.

*EjODO1* is an R2R3 type MYB transcription factor with conserved R2R3 domain (**Figure 2**). Further phylogenetic analysis indicated that *EjODO1* is different from previously characterized *EjMYB* genes (**Figure 3**), and it was closest to *PhODO1*, a transcriptional regulator of volatile benzenoids in petunia flowers (Verdonk et al., 2005) and two lignin biosynthesis and secondary cell-wall related transcription factors (*AtMYB85* and *AtMYB42*; Zhong et al., 2008).

### Association between *EjODO1* and Lignin Content in Different Loquat Tissues and Fruit Developmental Stages

**FIGURE 4:**
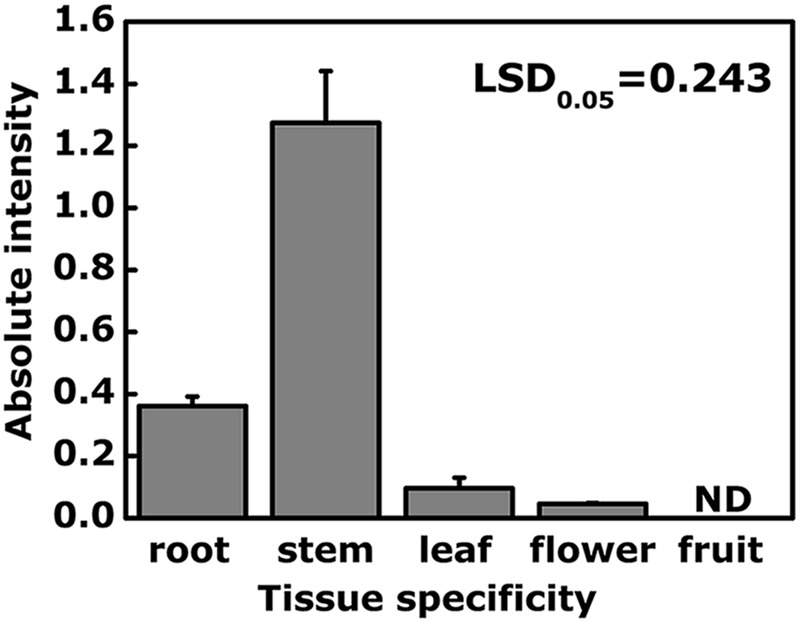
**Analysis of expression of *EjODO1* in different tissues of loquat.** Five tissues were used for gene expression of *EjODO1*, inculding vegetative tissues (roots, stems, and leaves of seedlings) and reproductive tissues (flowers, harvested at full bloom; fruit, mature fruit at harvest). Gene expression was expressed as absolute intensity compared to *EjACT*. Data are reported as means + SE. LSDs represent least significantdifference at 0.05. ND, not detectable in the pulp.

**FIGURE 5:**
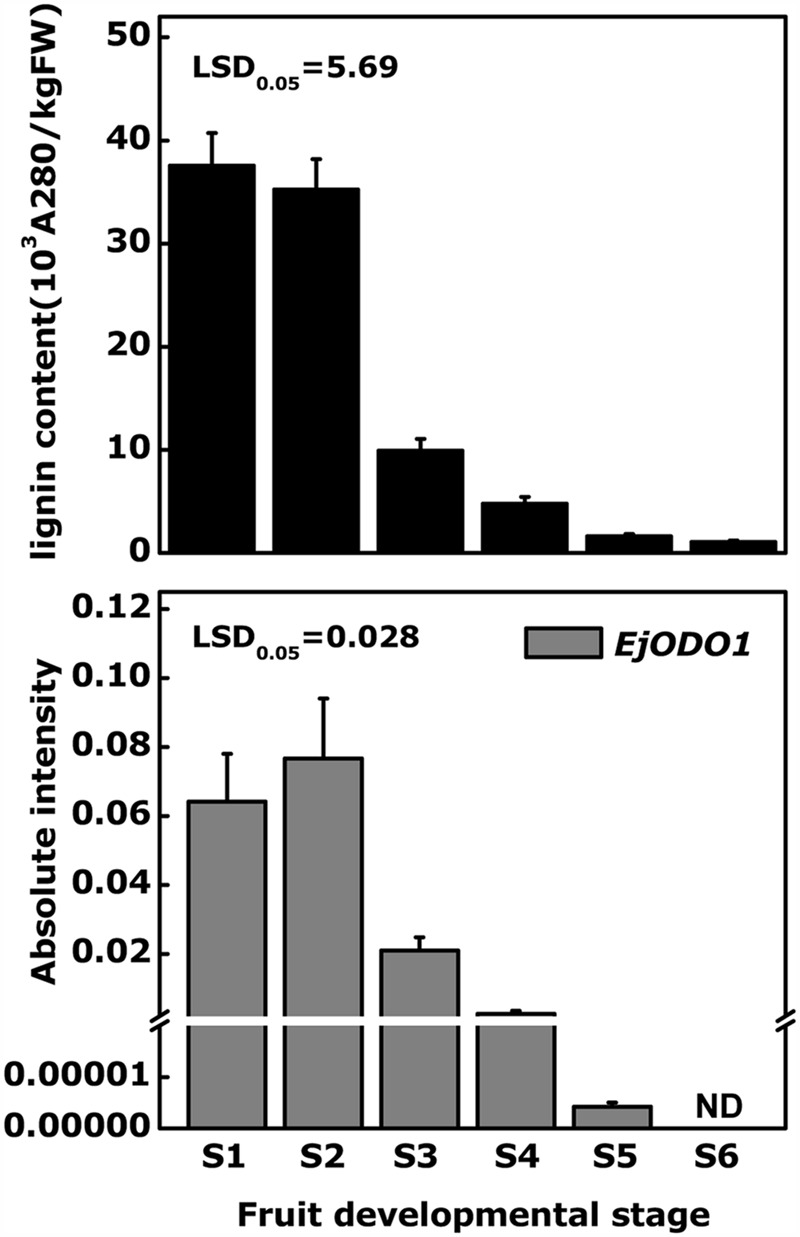
**Comparison of changes in lignin content and expression of *EjODO1* at different developmental stages of loquat fruit.** Red-flesh loquat ‘LYQ’ fruits were harvested at six different stages during fruit development:S1, 60 days afterfull bloom (DAFB); S2, 75 DAFB; S3,90 DAFB; S4, 100 DAFB; S5, 108 DAFB; S6, 115 DAFB, respectively. Fruit reach commercial mature at stage S6. Gene expression was expressed as absolute intensity compared to *EjACT*. Lignin content was expressed as A280 absorbance per kilogram of fresh weight (10^3^ A_280_ kg^-1^ of fresh weight). ND, not detected. Error bars indicate SEs from three biological replicates.

Tissue specificity analysis indicated that *EjODO1* was most highly expressed in stems and at a much lower level in roots, leaves and flowers, and was undetectable in the flesh of commercial mature loquat fruit (**Figure 4**). In order to investigate whether *EjODO1* is either not expressed at all, or only at a specific stage in fruit development, levels were examined in a loquat fruit developmental series. As shown in **Figure 5**, lignin content in loquat flesh gradually decreased during fruit development, from 37.57 × 10^3^A_280_kg^-1^FW^-1^ at S1 stage to 1.06 × 10^3^ A_280_kg^-1^FW^-1^at S6 stage (commercial maturity). Meanwhile, the expression level of *EjODO1* gradually decreased during fruit developmental, reaching an extremely low level at S5 stage (with approximate 0.0065% of S1 stage fruit) and was non-detectable at the S6 (ripe) stage (**Figure 5**). *EjODO1* also could not be detected in low temperature-induced lignified loquat fruit (data not shown).

### *In vivo* Interaction of EjODO1 and Promoters of Loquat Lignin Biosynthesis Genes

**FIGURE 6:**
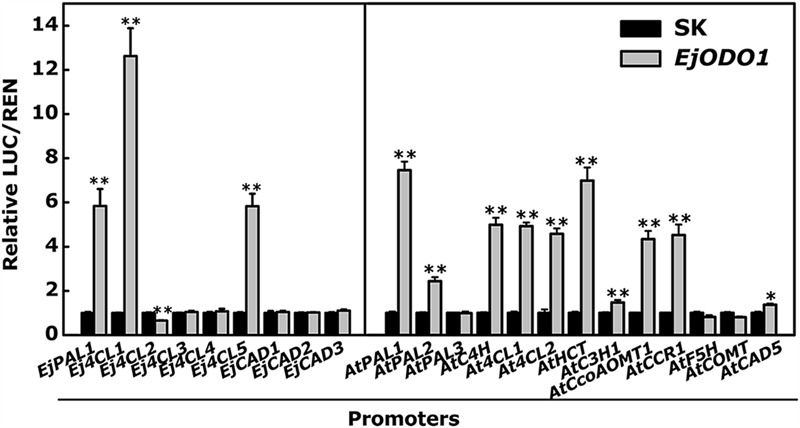
***In vivo* interaction of *EjODO1* with promoters of lignin biosynthesis genes from loquat and *Arabidopsis*.** 9 promoters of lignin biosynthesis from loquat and 13 promoters within the phenylpropanoid pathway from *Arabidopsis* were used for dual luciferase assey. The ratio of LUC/REN of the empty vector (SK) plus promoter was used as calibrator (set as 1). Error bars indicate SE from at least three replicates. Statistical analyses were performed using Student’s *t*-test: ^∗^*P* < 0.05, ^∗∗^*P* < 0.01.

Using the dual-luciferase assay, the trans-activation activities of *EjODO1* on the promoters of the *EjPAL, Ej4CL*, and *EjCAD* genes from loquat were investigated. The results indicated that *EjODO1* trans-activated the promoters of *EjPAL1, Ej4CL1*, and *Ej4CL5*, reached approximately 5.5, 11.5, and 5.7 fold, respectively (**Figure 6**). In order to further test the relationship between *EjODO1* and lignin biosynthesis, 13 genes within the phenylpropanoid pathway of *Arabidopsis* were selected for further analysis. The results showed that *EjODO1* could interact with many of the promoters of these lignin-related genes, including *AtPAL1, AtPAL2, AtC4H, At4CL1, At4CL2, AtHCT, AtC3H1, AtCCoAOMT1, AtCCR1*, and *AtCAD5* (**Figure 6**).

### Transient Expression of *EjODO1* and its Role in Lignin Biosynthesis

**FIGURE 7:**
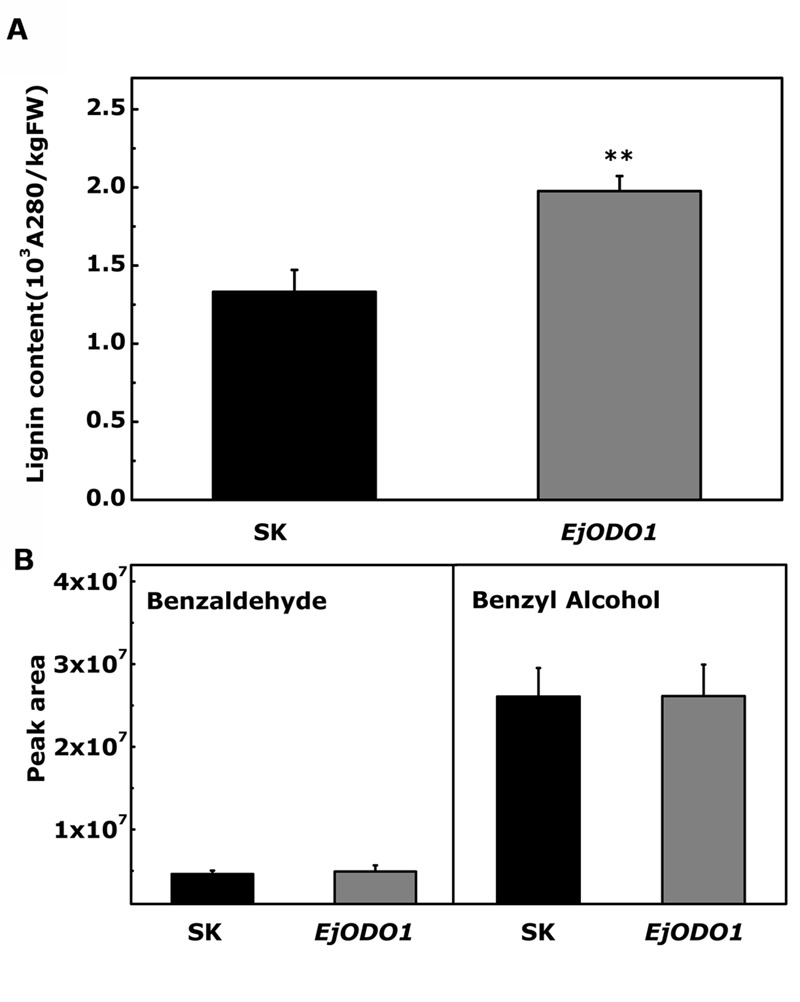
**Transient over-expression of *EjODO1* in *Nicotiana tabacum* leaves.** The transient over-expression experiments were conducted with empty vector pGreen II 0029 62-SK (SK) and *EjODO1* on two separate sides of the fifth leaf. Tissues from each of the infiltrated leaves were taken to measure both lignin content **(A)** and volatile compotents **(B)** after 5 days of infiltration. Lignin content was measured based on precipitation of lignin thioglycolic acid. Volatile compounds were determined by HS SPME GC-MS. Error bars indicate SEs from three biological replicates. Statistical analyses were performed using Student’s *t*-test: ^∗∗^*P* < 0.01.

In order to verify the significance of the results obtained from yeast one hybrid and dual-luciferase assay, transient over-expression was performed for functional analysis of *EjODO1* in tobacco leaves. *EjODO1*, driven by the CaMV 35S promoter in the pGreen II 0029 62-SK vector, was introduced into *N. tabacum* leaves using *Agrobacterium*. The results indicated that *EjODO1* significantly increased lignin content to 1.97 × 10^3^A_280_kg^-1^FW^-1^(*P* < 0.01), compared with 1.33 × 10^3^A_280_kg^-1^FW^-1^ for tobacco leaves expressing the empty vector (**Figure 7A**). With regard to the volatile analysis, two volatile compounds benzaldehyde and benzyl alcohol, products of the phenyl propanoid pathway were detected, but there were no differences in volatiles between the control (SK) and *EjODO1* treatments (**Figure 7B**).

## Discussion

Lignin biosynthesis and the process of lignification is regulated by many transcription factors, such as *EjMYB1, EjMYB2, EjMYB8, EjNAC1*, and *EjAP2-1*, which have been investigated in relation to the regulation of fruit lignification. Among these transcription factors, *EjMYB1, EjMYB2*, and *EjMYB8* could directly bind to and transcriptionally regulate promoters of lignin biosynthetic genes (Xu et al., 2014; Wang et al., 2016), while EjAP2-1 forms a protein-protein complex with EjMYB (Zeng et al., 2015), and the regulatory mechanism of *EjNAC1* remains unclear (Xu et al., 2015).

Here, a previously characterized fruit lignification-related gene, *Ej4CL1*, was selected for cDNA library screening, using yeast one hybrid assay, and *EjODO1* was identified. *EjODO1* has a conserved R2R3 domain and belongs to a member of the MYB family, which is similar to *EjMYB1, EjMYB2*, and *EjMYB8* (Wang et al., 2016; Xu et al., 2014). MYB proteins function as key regulators of the synthesis of phenylpropanoid-derived compounds, and some R2R3-MYB transcription factors regulate the synthesis of more than one class of phenylpropanoid-derived metabolites. Based on phylogenetic analysis, *EjODO1* clustered with *PhODO1* in petunia flowers, which is related to volatile benzenoids (Verdonk et al., 2005; Spitzer-Rimon et al., 2012) and *AtMYB85*, which was considered as lignin specific activator (Zhong et al., 2008). Overexpression of *VvMYB5a* in tobacco affected the metabolism of anthocyanins, flavonols, tannins, and lignins (Deluc et al., 2006). Moreover, *AtMYB75/PAP1* regulated a series of phenylpropanoid-derived compounds, including monolignol, anthocyanin, proanthocyanidin, flavonols, and phenolic acid (Deluc et al., 2006; Zuluaga et al., 2008). All these findings indicated the dual potential for *EjODO1* in lignin regulation and fragrance biosynthesis.

The expression pattern showed that *EjODO1* was highly and preferentially expressed in stems and roots of loquat, indicating that it may play a significant role in processes involved in formation of these actively lignifying tissues. These results were similar to some other previous observations with known MYB transcription factors, such as *AtMYB103*, which is only expressed in stems and influences the biosynthesis of S lignin in *Arabidopsis* stems (Zhong et al., 2008; Ohman et al., 2013); *AtMYB75* showed the highest expression in the lower part of the inflorescence stem, and lower levels of *AtMYB75* expression could be detected in flowers, leaves, and siliques, but none could be detected in roots (Bhargava et al., 2010). Moreover, the expression level of *EjODO1* diminished as fruit ripening proceeded, speculated to be involved in lignin biosynthesis during fruit development, especially for young fruit. *EjODO1* is not detectable in mature fruit and its subsequent storage period, which is different from previously characterized transcription factors, such as *EjMYB1, EjMYB2*, and *EjMYB8*, as these transcription factors were generally characterized as related to postharvest lignification in loquat fruit (Xu et al., 2014; Wang et al., 2016).

Further analysis indicated *EjODO1* is a transcriptional activator of lignin biosynthesis. Dual-luciferase assay revealed that *EjODO1* induced promoter activities of lignin biosynthesis genes in both loquat and *Arabidopsis*, which was similar to the transcriptional activator *EjMYB1* and functionally opposite to the transcriptional repressor *EjMYB2* (Xu et al., 2014). Other lignin-specific transcription factors, such as *AtMYB58, AtMYB63*, and *AtMYB85* are able to specifically induce expression driven by promoters of lignin biosynthesis genes, including promoters of *PAL* and *4CL* (Zhong et al., 2008; Zhou et al., 2009). Yeast one hybrid assay indicated that *EjODO1* could directly bind to the promoter of *Ej4CL1* and then promote trans-activation of the target genes. Transient overexpression of *EjODO1* was conducted to determine the lignin content and volatile substances, and the results indicated that *EjODO1* could promote the accumulation of lignin without any change in volatiles between the control (SK) and *EjODO1* expressers. Thus, it appears that in loquat, *ODORANT1* activity leads to transcriptional regulation of lignin production but not volatile benzenoid biosynthesis, which differ to its homolog in petunia *PhODO1* (Verdonk et al., 2005; Spitzer-Rimon et al., 2012).

## Conclusion

The present study has identified a novel activator of loquat lignin biosynthesis, *EjODO1*, by yeast one-hybrid screening, which, unlike the previously characterized transcription factors, appears to be a regulator of lignin biosynthesis in vegetative organs and during early fruit development.

## Author Contributions

JZ, X-rY, and K-sC designed the experiment. JZ, HG, and CZ performed the experiment. JZ and X-rY wrote the first draft of the manuscript. X-rY, XL, DG, and K-sC conducted analysis of data. X-rY, XL, DG, and K-sC contributed substantially to the revisions.

## Conflict of Interest Statement

The authors declare that the research was conducted in the absence of any commercial or financial relationships that could be construed as a potential conflict of interest.

## Footnotes

1 http://frodo.wi.mit.edu/primer3/

## Supplementary Material

FIGURE S1 **Protein-DNA interaction between the screened proteins and the promoter of *Ej4CL1* using yeast one hybrid analysis**.
