# Supplementary figures and images for: EjODO1, a MYB Transcription Factor, Regulating Lignin Biosynthesis in Developing Loquat (Eriobotrya japonica) Fruit
Source: Front Plant Sci. 2016 Sep 16;7:1360. doi: 10.3389/fpls.2016.01360 (PMC5025436; doi:10.3389/fpls.2016.01360)

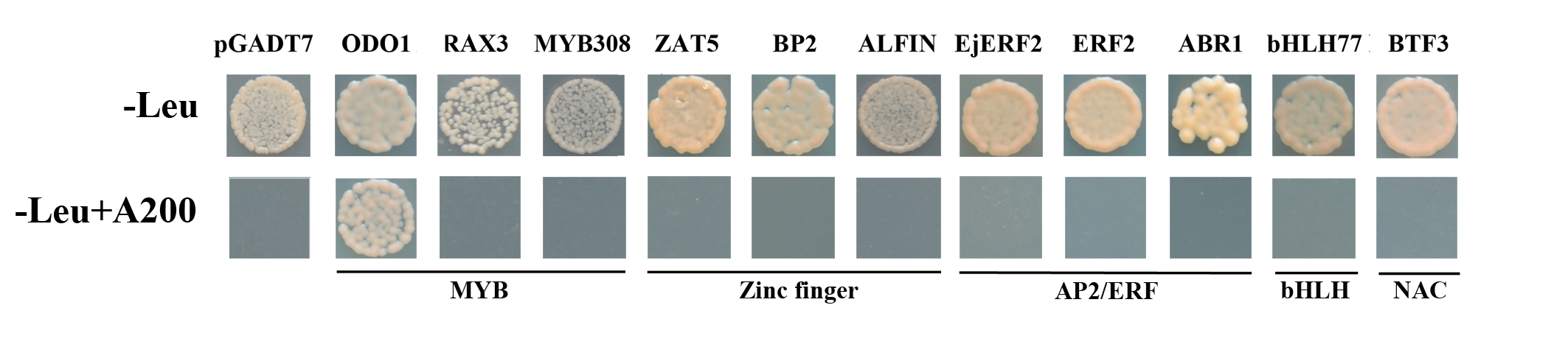

Supplement: FIGURE S1 — Protein-DNA interaction between the screened proteins and the promoter of Ej4CL1 using yeast one hybrid analysis. [file Image_1.TIF]
